# Composition and Diversity of the Ocular Surface Microbiota in Patients With Blepharitis in Northwestern China

**DOI:** 10.3389/fmed.2021.768849

**Published:** 2021-12-07

**Authors:** Changhao Wang, Xiuhong Dou, Jian Li, Jie Wu, Yan Cheng, Na An

**Affiliations:** ^1^College of Life Science, Northwest University, Xi'an, China; ^2^Department of Ophthalmology, Xi'an No.1 Hospital, First Affiliated Hospital of Northwest University, Xi'an, China; ^3^Shaanxi Key Laboratory of Ophthalmology, Shaanxi Provincial Clinical Research Center for Ophthalmic Diseases, Shaanxi Institute of Ophthalmology, Xi'an, China

**Keywords:** blepharitis, 16S rDNA amplicon sequencing, eyelid margin, conjunctival sac, microbiota

## Abstract

**Purpose:** To investigate the composition and diversity of the microbiota on the ocular surface of patients with blepharitis in northwestern China via 16S rDNA amplicon sequencing.

**Methods:** Thirty-seven patients with blepharitis divided into groups of anterior, posterior and mixed blepharitis and twenty healthy controls from northwestern China were enrolled in the study. Samples were collected from the eyelid margin and conjunctival sac of each participant. The V3–V4 region of bacterial 16S rDNA in each sample was amplified and sequenced on the Illumina HiSeq 2500 sequencing platform, and the differences in taxonomy and diversity among different groups were compared.

**Results:** The composition of the ocular surface microbiota of patients with blepharitis was similar to that of healthy subjects, but there were differences in the relative abundance of each bacterium. At the phylum level, the abundances of *Actinobacteria, Cyanobacteria, Verrucomicrobia, Acidobacteria, Chloroflexi*, and *Atribacteria* were significantly higher in the blepharitis group than in the healthy control group, while the relative abundance of *Firmicutes* was significantly lower (*p* < 0.05, Mann-Whitney U). At the genus level, the abundances of *Lactobacillus, Ralstonia, Bacteroides, Akkermansia, Bifidobacterium, Escherichia-Shigella, Faecalibacterium*, and *Brevibacterium* were significantly higher in the blepharitis group than in the healthy control group, while the relative abundances of *Bacillus, Staphylococcus, Streptococcus*, and *Acinetobacter* were significantly lower in the blepharitis group (*p* < 0.05, Mann-Whitney U). The microbiota of anterior blepharitis was similar to that of mixed blepharitis but different from that of posterior blepharitis. *Lactobacillus* and *Bifidobacterium* are biomarkers of posterior blepharitis, and *Ralstonia* is a biomarker of mixed blepharitis. There was no significant difference in the ocular surface microbiota between the eyelid margin and conjunctival sac with or without blepharitis.

**Conclusion:** The ocular surface microbiota of patients with blepharitis varied among different study groups, according to 16S rDNA amplicon sequencing analysis. The reason might be due to the participants being from different environments and having different lifestyles. *Lactobacillus, Bifidobacterium, Akkermansia, Ralstonia*, and *Bacteroides* may play important roles in the pathogenesis of blepharitis.

## Introduction

Blepharitis is defined as a chronic inflammatory condition of the eyelid margin associated with various discomforts including soreness, itching, tearing, irritation, and a burning sensation (1, 2). Long-term serious blepharitis may cause other ocular surface complications, such as dry eye, chalazion, eyelid ulcer, and blepharokeratoconjunctivitis (BKC), leading to severe ocular surface infection and even blindness (2, 3). The pathogenic mechanism of blepharitis is still under investigation, and the cause is generally believed to be a combination of infection (bacterial, viral, fungal or parasitic infection), immunity and metabolism (4–6). Bacteria are is an important etiologic factor in blepharitis, and many studies have shown that infection by pathogenic bacteria or destruction of the ecological balance of bacteria on the ocular surface may trigger blepharitis symptoms (7–9).

In past decades, several studies have investigated the ocular surface microbiota of blepharitis using culture approaches and found that *Staphylococcus epidermidis, Staphylococcus aureus, Corynebacterium macginleyi*, and *Propionibacterium acnes* were the most common species that appeared in cultures of eyelid margin or conjunctival sac samples from either patients with blepharitis or healthy controls (7–10). The relative differences in the prevalence of each bacterium were remarkable among the study groups from different parts of the world, which was believed to be due to the effects of different climates and environments (6, 11, 12). However, it is still doubtful whether the results of culture, which is susceptible to the influence of culture conditions, can reflect the true composition of the microbiota. In recent years, as a culture-independent approach with high sensitivity and specificity, high-throughput sequencing technology has been considered more appropriate to analyze the human microbiota than traditional culture methods, and the relative abundances of tens or even hundreds of times more bacteria can be identified (13). To date, studies of the microbiota of the ocular surface using high-throughput sequencing technology have been launched in eastern Asia. Lee et al. collected eyelash and tear samples from seven patients with blepharitis and four healthy people and analyzed the microbiota by 16S rDNA pyrosequencing (14). Yan et al. studied the microbiota of *Demodex* blepharitis from conjunctival sac samples by 16S rDNA pyrosequencing (15). Dong et al. investigated the composition of the bacterial community of patients with meibomian gland dysfunction (MGD) from conjunctival sac samples via 16S rDNA sequencing (16). However, there were some defects in the above studies, such as limited sample size, unsuitable sample type or different classifications. Each study was inadequate for a comprehensive analysis of the ocular surface microbiota of blepharitis.

In this study, we divided patients with blepharitis into anterior, posterior and mixed blepharitis groups and tried to explore the microbiome of both the conjunctiva and eyelid margin (including meibomian gland secretions) by 16S rDNA amplicon sequencing. This study is expected to provide a more specific reference to clarify the pathogenic mechanism of blepharitis.

## Materials and Methods

### Diagnosis of Blepharitis

The diagnosis of blepharitis was established according to the Blepharitis Preferred Practice Pattern of the American Academy of Ophthalmology (17). Blepharitis can be classified according to the anatomic location or underlying cause (1, 2, 17–19). (1) Anterior blepharitis is characterized by scabs at the eyelid margin, erythema and scales at the root of the eyelash. (2) Posterior blepharitis is characterized by loss of the meibomian gland, obstruction of meibomian gland duct orifices, foam on the eyelid margin, hypertrophy of the eyelid margin, trichiasis, and meibomian gland cyst formation. (3) Mixed blepharitis involves the characteristics of both the anterior and posterior eyelid margins.

### Ethics and Study Subjects

This study was approved by the Ethics Committee of Xi'an No. 1 Hospital (IRB: 2021–4) and registered on the International Clinical Trials Registry Platform (Registration number: ChiCTR2100042517). The study subjects were recruited from January to April 2021 at Xi'an No.1 Hospital after registration. In accordance with the tenets of the Declaration of Helsinki, written informed consent was obtained from each participant.

A total of 37 subjects with blepharitis were recruited and classified into the anterior blepharitis group (10), posterior blepharitis group (13), and mixed blepharitis group (14). The control group consisted of 20 subjects without any inflammation on the ocular surface, especially the eyelid margin. The characteristics of each group are shown in Table 1. The participants in the anterior blepharitis group were younger than those in any other group. There was a significant difference in age between the anterior blepharitis group and any other group (*p* < 0.05, Student's *t*-test) while there were no significant differences among the other three groups (*p* > 0.05, Student's *t*-test). The exclusion criteria were as follows: (1) patients who had used topical or systemic antibiotics within 1 month; (2) patients with a history of eye trauma or ophthalmic surgeries within 1 year; (3) pregnant or lactating women; (4) patients with systemic diseases affecting the ocular surface, such as Sjögren's syndrome or diabetes; and (5) patients with incomplete patient medical records.

**Table 1:** Characteristics of the participants in each group.

	**Healthy**	**Anterior**	**Posterior**	**Mixed**
	**control**	**blepharitis**	**blepharitis**	**blepharitis**
Number	20	10	13	14
Age, (mean ± SD)	59.16 ± 16.88	23.40 ± 15.53	51.77 ± 9.42	53.21 ± 18.90
Sex (female: male)	16:4	8:2	11:2	9:5

### Sample Collection

Swab samples from subjects in the blepharitis groups were taken from one eye with confirmed blepharitis, and those from the control group subjects were taken from one random eye. The subjects sat down and were asked to look up. After topical anesthesia with 0.4% oxybuprocaine hydrochloride eye drops (Santen, Osaka, Japan), a disposable aseptic cotton swab was used to wipe the lower conjunctival sac from the nasal to temporal side and backwards while rotating the swab. This process was repeated twice, avoiding sample contamination from the eyelashes or outer eyelid skin. Then, two sterile cotton swabs were used to squeeze the lower meibomian gland from the bottom to top both inside and outside of the lower eyelid until the meibum was visible at the openings, and the lower eyelid margin including the squeezed meibum was sampled from the nasal to temporal side and backwards. Each swab was immediately placed into a sterile tube and stored in an ultralow-temperature freezer at −80°C before DNA extraction.

### DNA Extraction, PCR Amplification and 16S RDNA Amplicon Sequencing

DNA was extracted from all samples using the MN NucleoSpin 96 Soi DNA Isolation Kit (MN-MACHEREY-NAGEL, Germany). The V3–V4 region of the 16S rRNA gene was amplified by PCR with the primers 338F (5'-ACTCCTACGGGAGGCAGCA-3'), and 806R (5'-GGACTACHVG GGTWTCTAAT-3'). The amplified products were purified, quantified and homogenized to form a sequencing library and sequenced on the Illumina HiSeq 2500 sequencing platform (Biomarker Technologies Corporation, Beijing, China) in PE250 mode (2 × 250 bp paired end sequencing) (Supplementary Material).

### Bioinformatics and Statistical Analysis

Bioinformatics analysis was conducted on the BMK Cloud Platform (www.biocloud.net). The raw reads were filtered by Trimmomatic (version 0.33), and then, Cutadapt (version 1.9.1), FLASH (version 1.2.7) and UCHIME (version 4.2) were used to obtain the effective reads. The effective reads with more than 97% identity were clustered into operational taxonomic units (OTUs) by USEARCH (version 10.0), while the OTUs whose proportions were <0.005% of the total OTUs were removed. Taxonomy was assigned using Silva (http://www.arb-silva.de) as the reference database, and the community composition of each sample was calculated at various levels (phylum, class, order, family, genus, and species). The alpha- and beta- diversities of all groups were obtained using QIIME2 and QIIME software, respectively. Linear discriminant analysis effect size (LEfSe) was used to identify bacterial biomarkers of each group. Student's *t*-test was used to compare the differences in age and sex between the patients with blepharitis and the controls. The Mann-Whitney *U*-test was performed for analyses of the α-diversity indices and the relative abundances of dominant phyla and genera among different groups. For principle coordinate analysis (PCoA), the permutational multivariate analysis of variance (PERMANOVA) statistical method was used to compare the differences. Statistical analyses were carried out with SPSS 26.0 (Chicago, IL, USA) software, and *p* < 0.05 was considered to be statistically significant.

## Results

### Bacterial Taxonomy

A total of 9,103,764 raw sequences were obtained from the 114 samples. After quality trimming and chimera checking, 8,697,646 high-quality sequences remained with an average of 76,295 high-quality sequences per sample for downstream analysis.

### Bacterial Alpha-Diversity in the Samples

**Figure 1:**
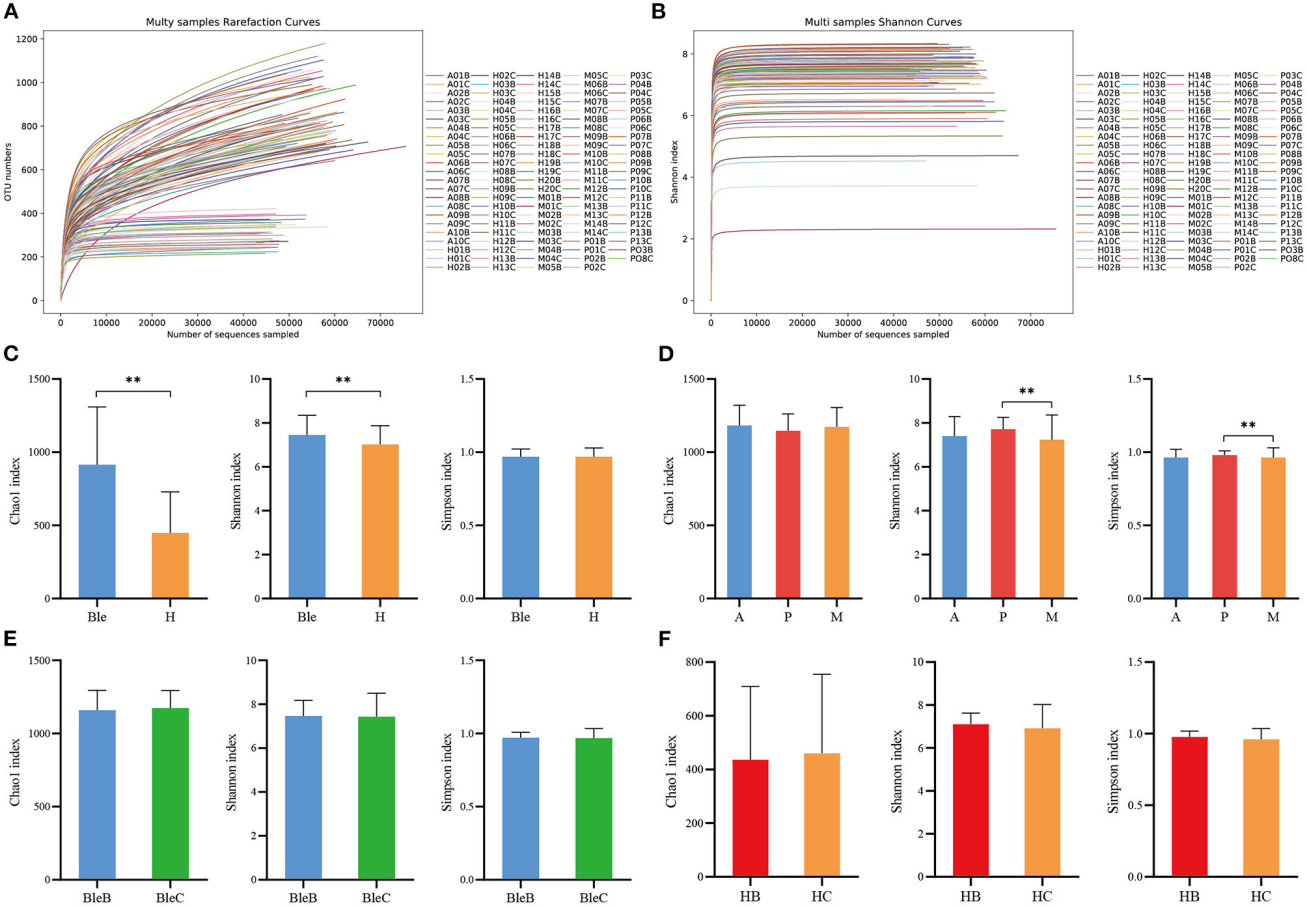
The α-diversity analysis of the samples. **(A)** Multisample rarefaction curves and **(B)** Shannon index curves. All rarefaction curves and Shannon index curves of the samples reached the saturation of the platform, indicating that the sequencing data size was adequate. **(C)** Comparisons of α-diversity indices between the blepharitis group (Ble) and healthy control group (H). **(D)** Comparisons of α-diversity indices among the anterior blepharitis group (A), posterior blepharitis group (P), and mixed blepharitis group (M). **(E)** Comparisons of α-diversity indices between the eyelid margin samples (BleB) and the conjunctival sac samples of the blepharitis group (BleC). **(F)** Comparisons of α-diversity indices between the eyelid margin samples (HB) and the conjunctival sac samples of the healthy control group (HC). ^**^P <0.01.

The richness (Chao1 index) and diversity (Shannon index and Simpson index) of the ocular surface microbiota within each group were calculated and evaluated. The rarefaction curves of the samples (Figure 1A) and the Shannon index curves (Figure 1B) reached saturation in all of the samples, which suggested that the species diversities discovered were adequate. The Chao 1 and Shannon indices were significantly higher in the blepharitis group than those in the healthy control group (*p* < 0.01, Mann-Whitney U), while the Simpson index was not significantly different between the two groups (*p* > 0.05, Mann-Whitney U) (Figure 1C). The Chao 1 index revealed no significant differences among the three subgroups of blepharitis (*p* > 0.05, Mann-Whitney U), while the Shannon index and Simpson index were significantly different between the posterior blepharitis group and mixed blepharitis group (*p* < 0.01, Mann-Whitney U) (Figure 1D). There were no significant differences in the Chao1 index, Simpson index or Shannon index between the eyelid margin samples and the conjunctival sac samples in either the blepharitis or healthy control group (*p* > 0.05, Mann-Whitney U) (Figures 1E,F).

### Bacterial Beta-Diversity in Samples

**Figure 2:**
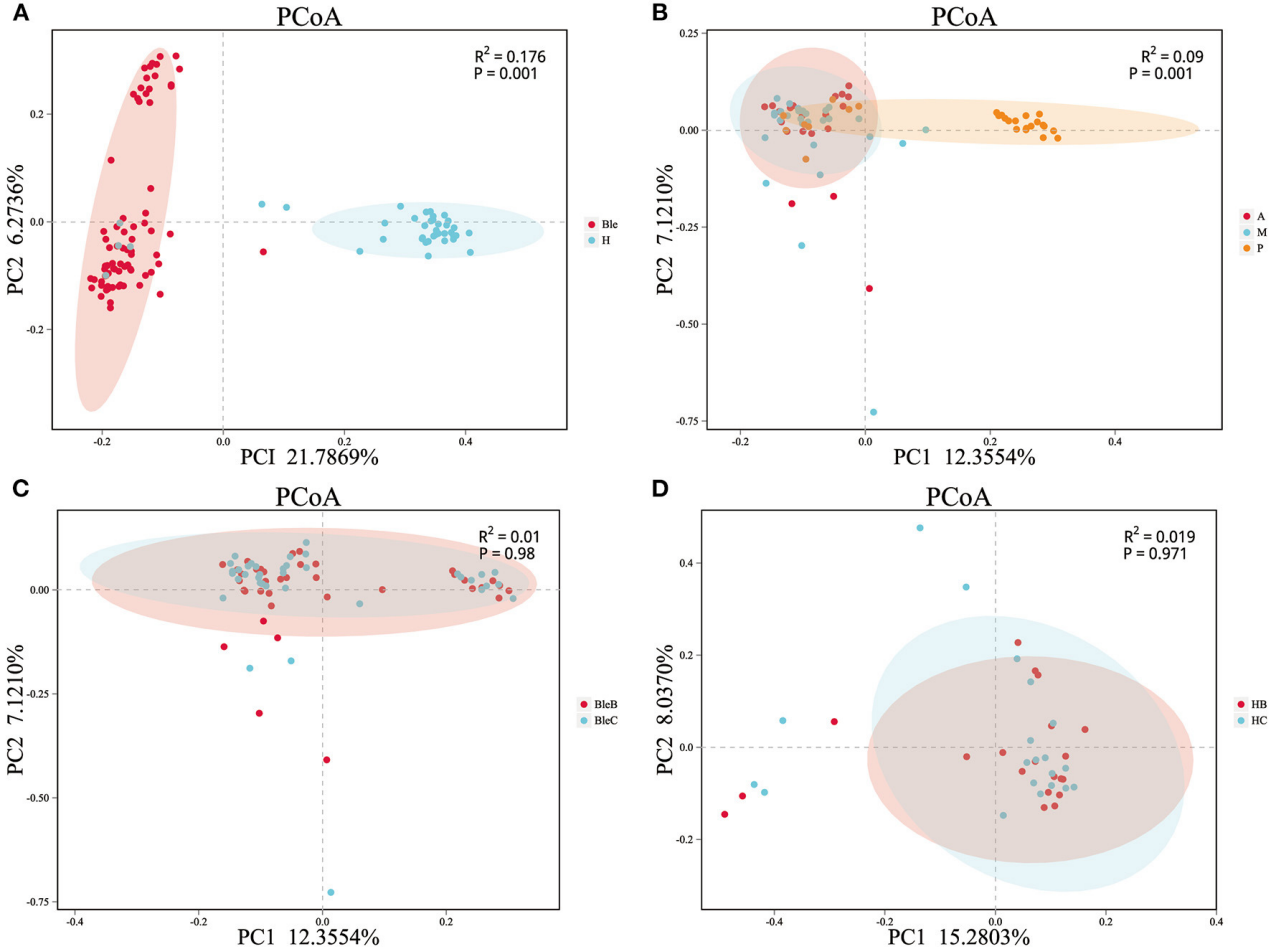
The β-diversity analysis of the samples. PCoA and PERMANOVA were performed using the Bray-Curtis method. Each dot corresponds to a sample, and each color represents a group. **(A)** Division of all of the samples into the blepharitis group (Ble) and healthy control group (H). **(B)** The samples of patients with blepharitis were divided into the anterior blepharitis group (A), mixed blepharitis group (M) and posterior blepharitis group (P). **(C)** The samples of the patients with blepharitis were divided into the eyelid margin of the blepharitis group (BleB) and the conjunctival sac of the blepharitis group (BleC). **(D)** The samples of the normal subjects were divided into the eyelid margin of the healthy control group (HB) and the conjunctival sac of the healthy control group (HC).

The results of beta-diversity analysis among different groups are shown by principal coordinate analysis (PCoA) and permutational multivariate analysis of variance (PERMANOVA). The PCoA indicated that the samples with high community structure similarity clustered, while when the samples were far from each other, a large difference in community structure was indicated. PERMANOVA can be used to test whether there are significant differences in beta diversity between the samples in different groups. Most of the clustered plots of each group indicated a significant difference in the ocular surface microbiota between the blepharitis and healthy control groups (Figure 2A). According to the division of the blepharitis group into three different types, the samples from the posterior blepharitis group were far apart from those from the anterior and mixed blepharitis groups; inversely, the samples from the anterior and mixed blepharitis groups were similar (Figure 2B). PERMANOVA indicated significant differences in beta diversity among the samples from the three groups (Figure 2B). Comparing the eyelid margin samples with the conjunctival sac samples of the blepharitis or healthy control group, most of the plots showed similar microbiota compositions, and there was no significant difference (Figures 2C,D).

### Bacterial Taxonomy in the Microbiota Composition of the Samples

**Figure 3:**
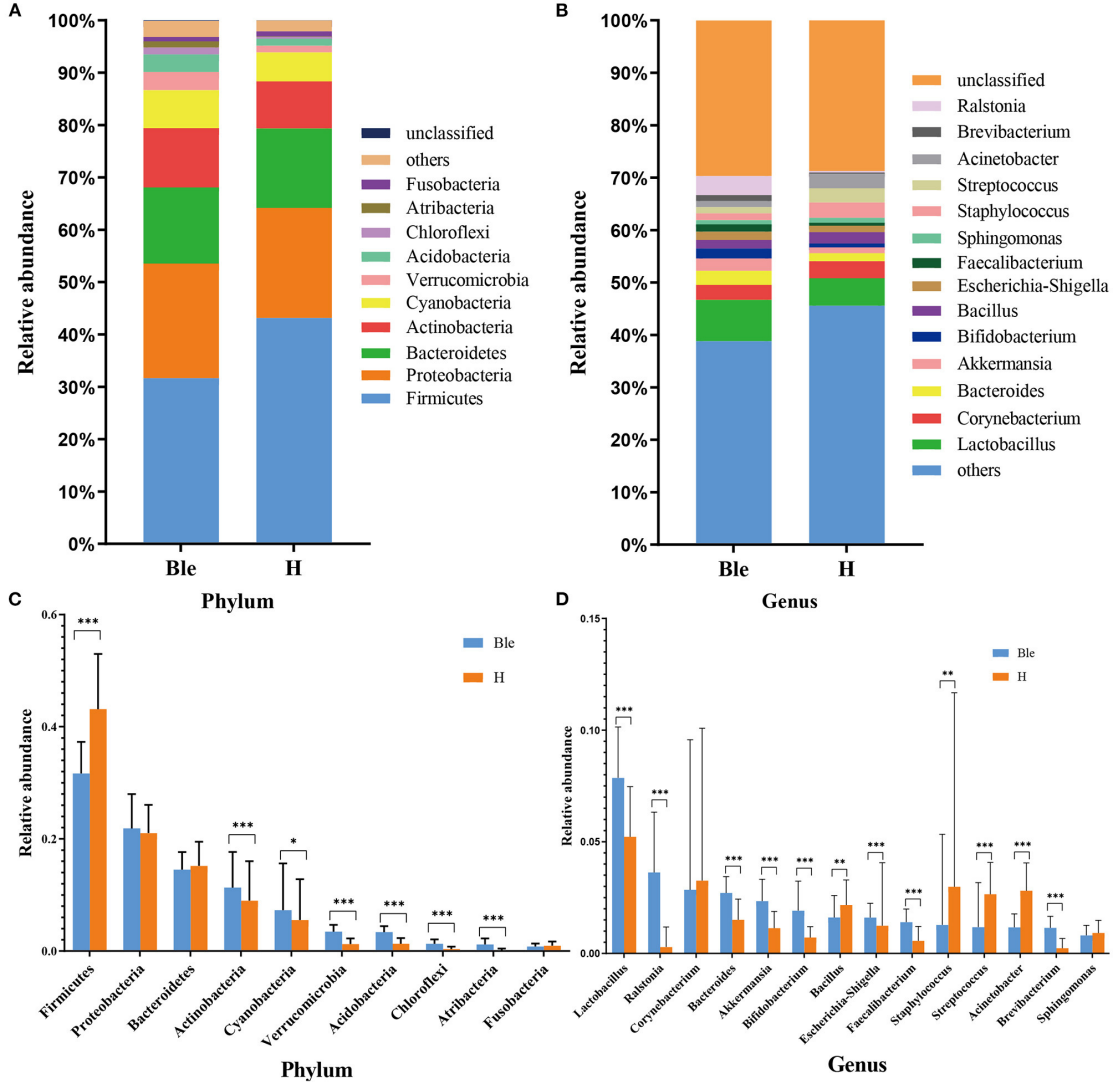
Differences in microbiota composition between the blepharitis group and healthy control group. **(A, B)** Each phylotype (1% average relative abundance in groups) is indicated by a different color at the phylum and genus levels. **(C, D)** At the phylum or genus level, there was a significant difference in the abundance of ocular surface microbiota between the blepharitis group (Ble) and the healthy control group (H). ^*^P < 0.05, ^**^P <0.01, ^***^P <0.001.

The relative abundances of the dominant ocular surface microbiota in the patients with blepharitis and the healthy controls were summarized. At the phylum level (Figure 3A), 31 phyla were detected in the 57 eyes. The major phyla in the subjects in the two groups included *Firmicutes* (31.67; 43.14%), *Proteobacteria* (21.89; 21.04%), *Bacteroidetes* (14.52; 15.17%), *Actinobacteria* (11.32; 9.00%), *Cyanobacteria* (7.30; 5.54%), *Verrucomicrobia* (3.45; 1.26%), *Acidobacteria* (3.37; 1.32%), *Chloroflexi* (1.29; 0.37%), *Atribacteria* (1.19; 0.10%), and *Fusobacteria* (0.81; 0.95%). At the genus level (Figure 3B), the most predominant bacteria were *Lactobacillus* (7.86; 5.22%), *Ralstonia* (3.63; 0.29%), *Corynebacterium* (2.85; 3.26%), *Bacteroides* (2.71; 1.51%), *Akkermansia* (2.34; 1.13%), *Bifidobacterium* (1.91; 0.71%), *Bacillus* (1.61; 2.17%), *Escherichia-Shigella* (1.61; 1.24%), *Faecalibacterium* (1.40; 0.56%), *Staphylococcus* (1.28; 2.98%), *Streptococcus* (1.18; 2.65%), *Acinetobacter* (1.16; 2.81%), *Brevibacterium* (1.15; 0.24%), and *Sphingomonas* (0.81; 0.92%).

### Differences Between Blepharitis Patients and Healthy Controls

At the phylum level (Figure 3C), the abundance of *Firmicutes* (*p* < 0.01, Mann-Whitney U) in the blepharitis group was significantly lower than that in the healthy control group, while the relative abundances of *Actinobacteria, Cyanobacteria, Verrucomicrobia, Acidobacteria, Chloroflexi*, and *Atribacteria* (*p* < 0.05, Mann-Whitney U) were significantly higher. The relative abundances of *Proteobacteria, Bacteroidetes*, and *Fusobacteria* in the blepharitis group were not significantly different from those in the healthy control group (*p* > 0.05, Mann-Whitney U). At the genus level (Figure 3D), compared with the healthy control group, the blepharitis group had a significantly higher abundance of *Lactobacillus, Ralstonia, Bacteroides, Akkermansia, Bifidobacterium, Escherichia-Shigella, Faecalibacterium*, and *Brevibacterium* (*p* < 0.01, Mann-Whitney U), and lower relative abundances of *Bacillus, Staphylococcus, Streptococcus* and *Acinetobacter* (*p* < 0.05, Mann-Whitney U). There were no significant differences in the relative abundances of *Corynebacterium* and *Sphingomonas* between the two groups (*p* > 0.05, Mann-Whitney *U*-test).

**Figure 4:**
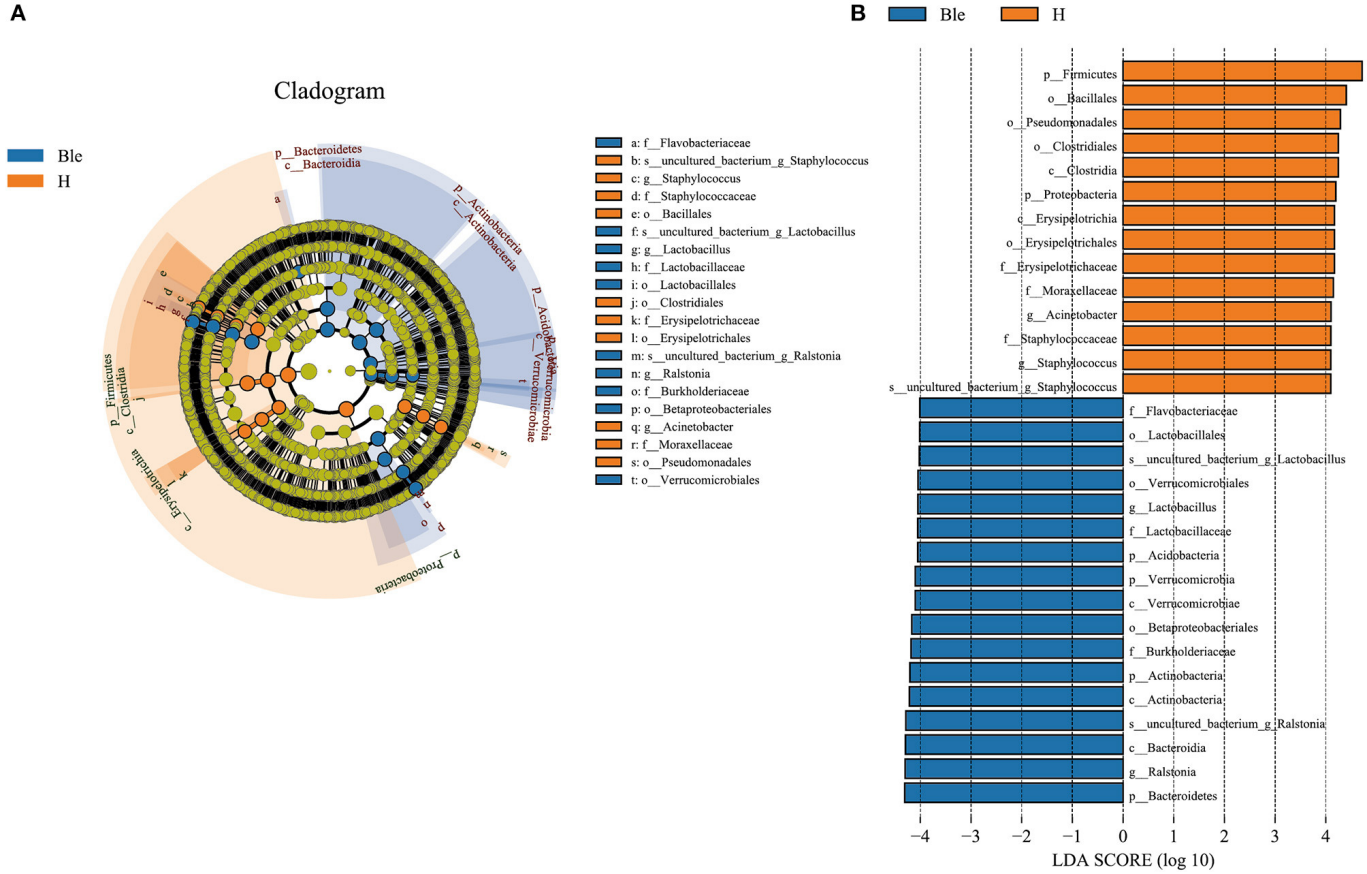
LEfSe analysis of the blepharitis group and healthy control group. **(A)** A cladogram of the ocular surface microbial taxa in the blepharitis (Ble) and healthy control groups (H) showing the levels from phylum to species from outside to inside. **(B)** Linear discriminant analysis (LDA) scoring of biomarkers corresponding to (A), computed by the LEfSe tool. When the score of a taxon was >4.0 with *P* < 0.01, it was listed in the histogram.

To determine the potential bacterial biomarkers between the blepharitis and healthy control groups, LEfSe at the phylum and genus levels (LDA >4, *p* < 0.01) was used. The biomarkers were *Bacteroidetes* (phylum), *Actinobacteria* (phylum), *Verrucomicrobia* (phylum), *Acidobacteria* (phylum), *Betaproteobacteriales* (order), *Burkholderiaceae* (family), *Flavobacteriaceae* (family), *Lactobacillus* (genus) and *Ralstonia* (genus) in the blepharitis group, *Firmicutes* (phylum), *Proteobacteria* (phylum), *Clostridia* (class), *Bacillales* (order), *Pseudomonadales* (order), *Erysipelotrichaceae* (family), *Moraxellaceae* (family), *Acinetobacter* (genus), and *Staphylococcus* (genus) in the healthy control group (Figures 4A,B).

### Differences in Microbiota Composition Between Anterior, Posterior and Mixed Blepharitis

**Figure 5:**
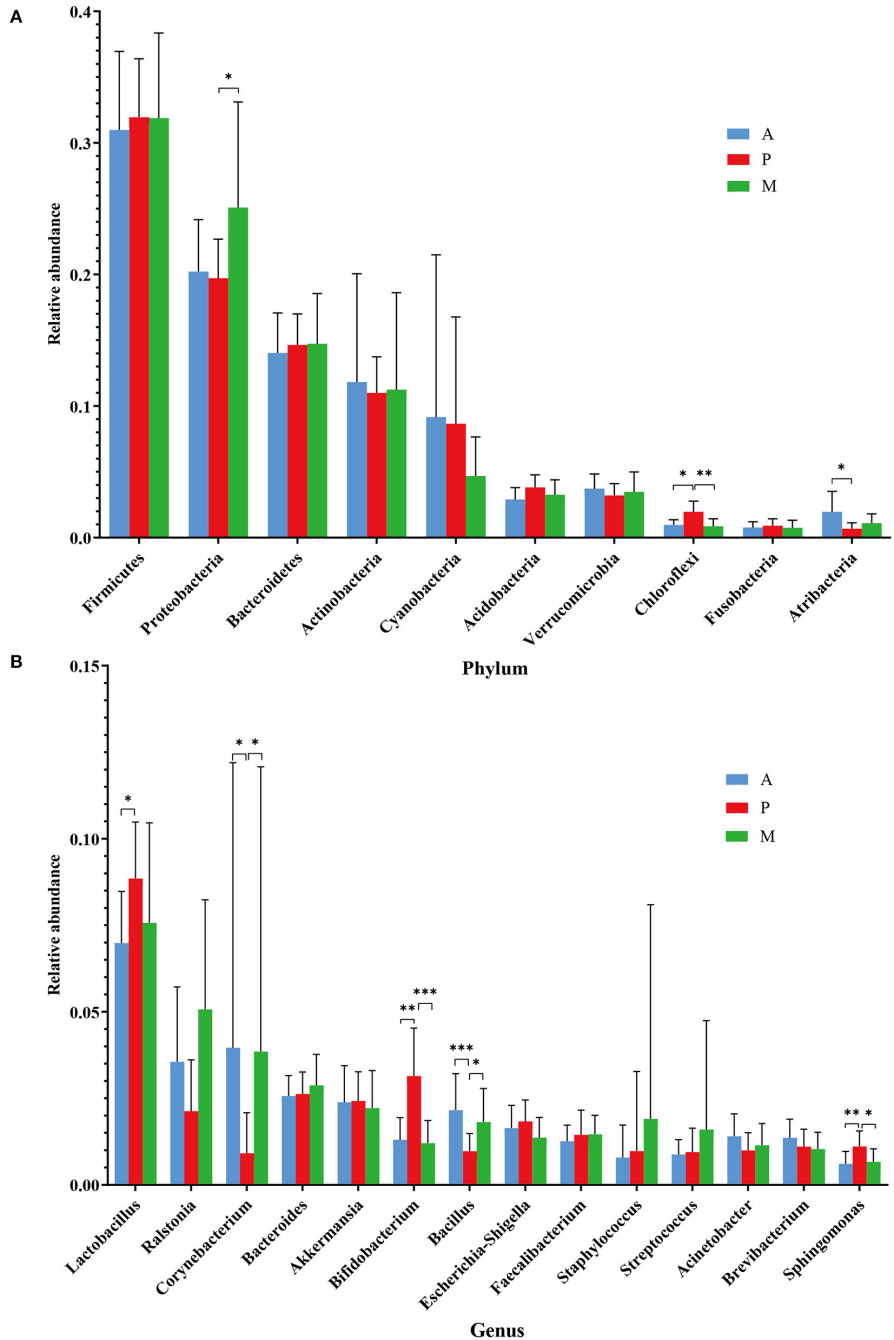
Differences in microbiota composition in the four groups. **(A, B)** At the phylum and genus levels, there were significant differences in the abundances of ocular surface microbiota among the anterior blepharitis group (A), posterior blepharitis group (P) and mixed blepharitis group (M). ^*^P <0.05, ^**^P <0.01, ^***^P <0.001.

All of the participants with blepharitis were divided into anterior, posterior and mixed groups. At the phylum level (Figure 5A), the posterior blepharitis group had a significantly higher abundance of *Chloroflexi* than the anterior blepharitis group (*p* < 0.05, Mann-Whitney U) or mixed blepharitis group (*p* < 0.01, Mann-Whitney U), but had a significantly lower abundance of *Atribacteria* than the anterior blepharitis group (*p* < 0.05, Mann-Whitney U). The mixed blepharitis group had a significantly higher abundance of *Proteobacteria* than the posterior blepharitis group. At the genus level (Figure 5B), the posterior blepharitis group had significantly higher abundances of *Lactobacillus, Bifidobacterium*, and *Sphingomonas* than the anterior blepharitis group or mixed blepharitis group (*p* < 0.05, Mann-Whitney U), but significantly lower abundances of *Corynebacterium* and *Bacillus* than the anterior blepharitis group or mixed blepharitis group (*p* < 0.05, Mann-Whitney U). There was no difference in the abundance of microbiota constituents between the anterior blepharitis group and the mixed blepharitis group.

**Figure 6:**
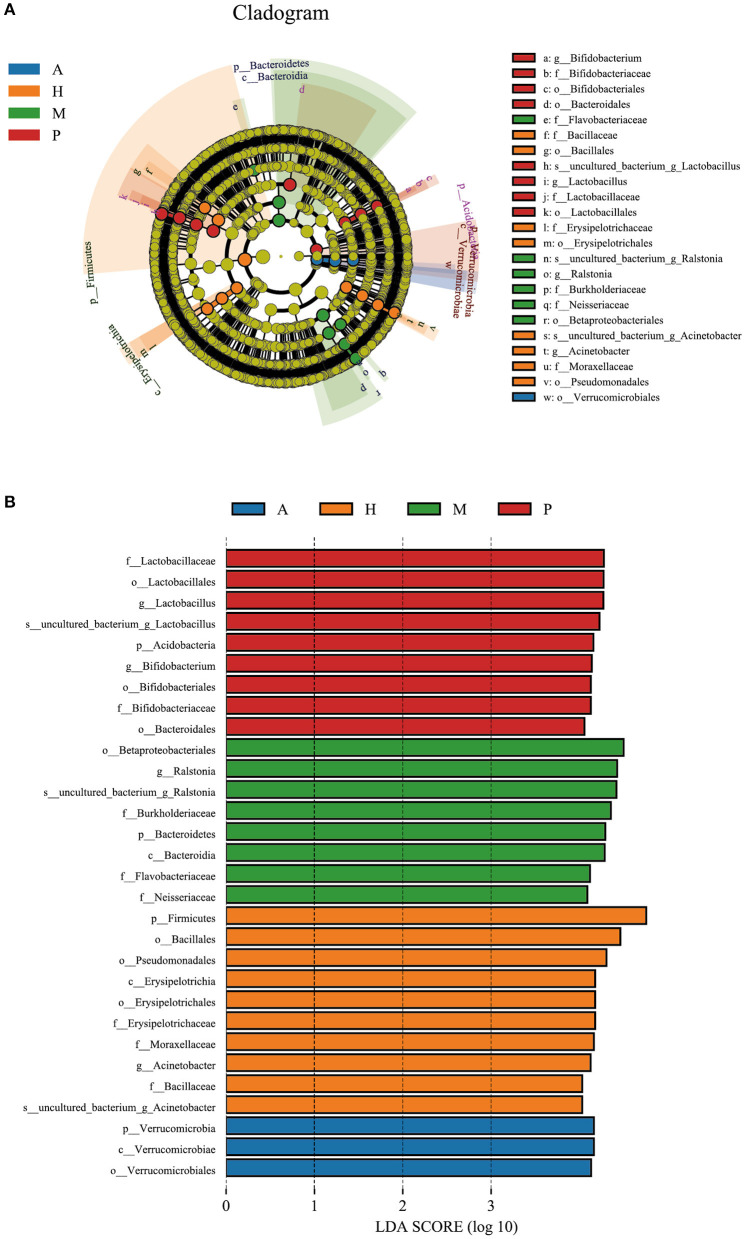
LEfSe of the four groups. **(A)** A cladogram of the ocular surface microbial taxa in the anterior blepharitis group (A), posterior blepharitis group (P), mixed blepharitis group (M) and the healthy control group (H). The levels from phylum to species from outside to inside. **(B)** Linear discriminant analysis (LDA) scoring of biomarkers corresponding to (A), computed by the LEfSe tool. When the score of a taxon was >4.0 with *p* < 0.01, it was listed in the histogram.

*Verrucomicrobia* (phylum) was the biomarker in the anterior blepharitis group. *Acidobacteria* (phylum), *Bacteroidetes* (order), *Lactobacillus* (genus), and *Bifidobacterium* (genus) were the biomarkers in the posterior group. *Bacteroidetes* (phylum), *Betaproteobacteriales* (order), *Burkholderiaceae* (family), *Flavobacteriaceae* (family), *Neisseriaceae* (family), and *Ralstonia* (genus) were the biomarkers in the mixed group. *Firmicutes* (phylum), *Pseudomonadales* (order), *Erysipelotrichaceae* (family), *Moraxellaceae* (family), *Bacillaceae* (family), and *Acinetobacter* (genus) were the biomarkers in the healthy control group (Figures 6A,B).

### Differences in Microbiota Composition Between the Conjunctival Sac and Eyelid Margin

**Figure 7:**
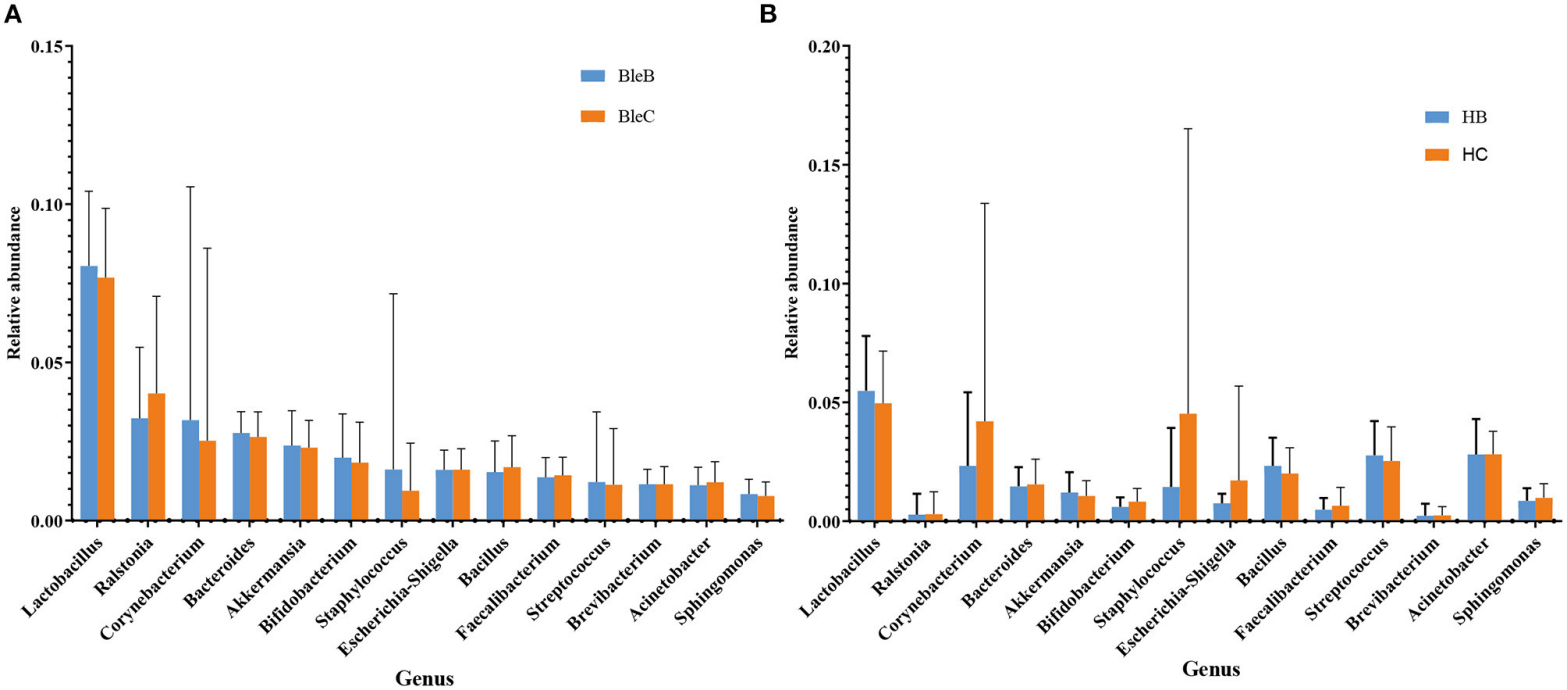
Differences in microbiota composition between the conjunctival sac and eyelid margin. **(A)** Comparison within the blepharitis group. **(B)** Comparison within the healthy control group.

The distribution characteristics of microbiota between the eyelid margin and conjunctival sac samples of the blepharitis group (Figure 7A) and the healthy control group (Figure 7B) at the genus level were also compared. There was no significant difference in the microbiota between either of the two groups (*p* > 0.05, Mann-Whitney U).

## Discussion

Blepharitis is a common ophthalmic disease that may be related to alterations in the ocular surface microbiota. It is of great clinical significance to study the composition of the ocular surface microbiota between blepharitis patients and healthy controls to elucidate the pathogenesis of blepharitis and formulate suitable treatments. Based on traditional culture methods, many studies have shown significant differences in the ocular surface microbiota between patients with and without blepharitis. These studies have shown that the most common bacteria in patients with blepharitis were *Staphylococcus epidermis, Staphylococcus aureus, Corynebacterium sp., Acinetobacter sp., Propionibacterium acnes*, and *Streptococcus viridans* (7–10). However, there have also been reports of conflicting results. For example, Groden et al. showed that *Staphylococcus aureus* was more common in the normal control group (8). Bezza et al. reported that the abundance of *Propionibacterium acnes* was almost the same in the blepharitis group and the normal control group (9). The differences between these studies were considered to be related to differences in environmental and culture conditions (6, 11, 12).

With the emergence and development of high-throughput sequencing technology, the rapid development of ocular surface microbiota research has been promoted. This line of research no longer limited by culture conditions, and dozens or even hundreds of species can be detected from a small sample amount, allowing the composition of the microbiota and the abundance of each species to be more truly reflected. *Corynebacterium, Pseudomonas, Propionibacterium, Acinetobacter, Streptococcus*, and *Anaerococcus* were the most abundant genera on the ocular surface microbiota of healthy humans. The abundances of *Staphylococcus, Propionibacterium*, and *Micrococcus* were not as great as those determined via culture, and some uncultured bacteria, such as *Streptophyta, Methylobacterium, Enhydrobacter*, and *Veillonella* were identified (11, 13, 20–24).

The study of the ocular surface microbiota of blepharitis was started by Lee et al., who found over 24 bacterial genera in eyelash and tear samples from seven patients and four healthy controls. At the genus level, *Propionibacterium, Staphylococcus, Streptophyta, Corynebacterium* and *Enhydrobacter* were common ocular bacteria in all the samples, and subjects with blepharitis had a lower proportion of *Propionibacterium* and higher *Staphylococcus, Streptophyta, Corynebacterium* and, *Enhydrobacter* abundances (14). Our study demonstrated that the ocular surface microbiota of patients with blepharitis was similar to that of healthy subjects, but there were differences in the relative abundance of each bacterium. Regardless of the occurrence of blepharitis, *Lactobacillus, Ralstonia, Corynebacterium, Bacteroides, Akkermansia, Bifidobacterium, Bacillus, Escherichia-Shigella, Faecalibacterium, Staphylococcus, Streptococcus, Acinetobacter, Brevibacterium* and *Sphingomonas* were the main ocular surface microbiota constituents in both conjunctival sac and eyelid margin samples (Figure 3). As biomarkers of the blepharitis group, *Lactobacillus* and *Ralstonia* were significantly more abundant in patients with blepharitis, as were some other genera such as *Bacteroides, Akkermansia*, and *Bifidobacterium*. *Lactobacillus* and *Bifidobacterium* are both popular gut probiotics that induce regulatory T cells and *Akkermansia, Bacteroides*, and *Ralstonia* are all common in gut microbiota (25–28). We hypothesized that they play a similar important role in the mucosal tissue on the ocular surface as that in the intestinal tract. The relative abundances of *Lactobacillus* and *Bifidobacterium* increased in patients with blepharitis, which may be a compensatory reaction to temporarily resist the invasion of pathogenic bacteria (29). *Akkermansia* has been found to be a mucin-engulfing bacterium that can produce acetate and propionate in the process of mucin degradation, which may have some anti-inflammatory effects (30–32). Recent studies have demonstrated that *Akkermansia* could enhance tight junctions and decrease intestinal epithelial layer permeability by excreting extracellular vesicles (33). *Bacteroides* is an opportunistic pathogen that can invade the submucosa and cause infection when the mucosa is damaged. *Bacteroides* can produce many metabolites such as short-chain fatty acids (SCFAs) and polysaccharide A (PSA), to influence metabolism (33, 34). *Ralstonia* is also a prevalent opportunistic pathogen that has recently been found in polluted city water systems. It may infect the eyelid margin during face washing and colonize the ocular surface. (35–39) The abundances of *Staphylococcus* and *Acinetobacter* were extremely elevated in healthy subjects, indicating that blepharitis has an effect on the abundances of *Staphylococcus* and *Acinetobacter*, suggesting that *Staphylococcus* and *Acinetobacter* might be more important in maintaining the health of the eyelid margin and conjunctival sac.

Dividing the blepharitis group into anterior, posterior and mixed blepharitis groups, we found that the microbiota of anterior blepharitis was similar to that of mixed blepharitis but different from that of posterior blepharitis. The abundances of *Lactobacillus, Bifidobacterium*, and *Sphingomonas* (especially the first two) were significantly higher in posterior blepharitis than in anterior or mixed blepharitis, and those of *Corynebacterium* and *Bacillus* were significantly lower in posterior blepharitis. The results confirmed that the pathogenesis of anterior and posterior blepharitis might be different, and the effects on different bacterial genera were also different. The anterior eyelid margin is close to the eyelid skin attached to eyelashes, while the posterior eyelid margin is adjacent to the conjunctiva of the eyelid and located at the opening of the meibomian glands. Anterior blepharitis affects the base of the eyelashes, and bacterial, seborrheic and *Demodex folliculorum* factors are the most likely causes, whereas posterior blepharitis is often associated with composition changes in meibum (4–6). Therefore, the differences in the compositions of the ocular surface microbiota might be due to different local physiological environments and relate to different pathogenic mechanisms, which suggests that it is necessary to strictly define different types of blepharitis for future research on the pathogenesis. The abundance of *Ralstonia* is extremely elevated in mixed blepharitis. Considering that *Ralstonia* is a popular opportunistic pathogen, it might reveal the greater severity of mixed blepharitis compared to pure anterior or posterior blepharitis.

Although there were several studies on the ocular surface microbiota of simple anterior (*Demodex*) blepharitis or posterior blepharitis (MGD) using 16S rDNA amplicon sequencing, the results showed both similar and contradictory compositions and changes. For example, Dong et al. and Zhao et al. both reported *Sphingomonas* as a biomarker of MGD, which was the same as our result. However, Dong et al. found that the abundance of *Corynebacterium* significantly decreased in patients with MGD, whereas Zhao et al. found that it increased (16, 40) Yan et al. found *Lactobacillus* to be the biomarker of *Demodex* blepharitis, which is different from our results (15). These conflicting results indicate that the complexity of individual ocular surface microbiota diversity and its influencing factors are far beyond our knowledge. To explain the different results among different studies, the most reasonable explanation might be the influence of the environment, such as climate and lifestyle. Using metagenomic analysis, Deng et al. found that geographical differences and environmental changes shaped the ocular surface microbiota, which is in accordance with the results regarding the blepharitis microbiota from different parts of the world (41). Considering that the eyelid margin is a special site adjacent to eyelid skin and has a similar anatomical structure and function, we could derive information from research on the skin microbiota. The composition of skin microbiota is an individual trait that depends on age, sex, hygiene habit, host lifestyle, and environment, which is determined by the skin structure, including the thickness of the skin, the depth and location of folds and the density of follicles and glands, and is also affected by external environmental conditions, including temperature, humidity, and sunlight, especially UV radiation. For example, *Corynebacterium* and *Staphylococcus* are dominant in humid regions and the microbiotas of individuals living in dry regions are not microbiologically diverse. Eccrine glands prevent the colonization and proliferation of microorganisms by water and electrolyte excretion and skin acidification, whereas the sebum secreted by the sebaceous glands is the source of lipids and free fatty acids, which facilitate the adhesion of bacteria and inhibit the growth of pathogens (42, 43). The participants in our study mainly came from northwestern China, where the drier temperate continental climate is different from the humid climate in coastal areas. Therefore, the results of our study may be quite different from those of the other study groups but are still of importance for research on the pathogenesis of blepharitis globally.

In our study, we also compared the microbiota of the eyelid margin and conjunctival sac. There was no significant difference between the two sites in either the blepharitis or healthy control group. The results revealed that the microbiota of the eyelid margin and conjunctiva were influenced by each other, which could explain why blepharitis tends to cause ocular surface inflammation such as conjunctivitis, keratitis, and even postoperative infection after inner eye surgery such as cataract surgery. It also reminds clinicians to pay attention to the health of eyelid margins when diagnosing and treating ocular surface inflammation or before intraocular surgery (44–47).

Unfortunately, our 16S rDNA amplicon sequencing analysis of blepharitis microbiota using universal primers for V3–V4 of 16S rDNA cannot identify the bacteria at the species level, which is what we need to improve the method in future studies. Sometimes, different species of the same genus may play different roles in such body sites of humans, for example, *Staphylococcus aureus, Staphylococcus epidermis, Streptococcus pneumoniae*, and *Streptococcus viridis*. The functions of the most important bacteria discovered in our study, such as *Lactobacillus, Ralstonia, Bacteroides, Akkermansia*, and *Bifidobacterium*, need to be proven at the species level. If we could improve our culture conditions to obtain a positive culture of such fastidious bacteria, we may acquire more information from such special strains. Furthermore, considering the tremendous individual diversity of the ocular microbiota, further studies with larger sample sizes and more specific classification designs are needed to elucidate the pathogenesis of blepharitis.

## Data Availability Statement

The datasets presented in this study can be found in online repositories. The names of the repository/repositories and accession number(s) can be found below: https://www.ncbi.nlm.nih.gov/, PRJNA744578.

## Ethics Statement

The studies involving human participants were reviewed and approved by Ethics Committee of Xi'an No. 1 Hospital. Written informed consent to participate in this study was provided by the participants' legal guardian/next of kin.

## Author Contributions

CW, NA, YC, and JW contributed to conception and design of the study. CW and XD carried out the experiment and organized the database. CW and JL performed the statistical analysis. CW wrote the first draft of the manuscript. NA and YC wrote sections of the manuscript. All authors contributed to manuscript revision, read, and approved the submitted version.

## Funding

This work was supported by the Shaanxi Provincial Department of Science and Technology (2021SF-331). Xi'an Municipal Science and Technology Bureau (Award numbers: 21YXYJ0027, 21YXYJ0002).

## Conflict of Interest

The authors declare that the research was conducted in the absence of any commercial or financial relationships that could be construed as a potential conflict of interest.

## Publisher's Note

All claims expressed in this article are solely those of the authors and do not necessarily represent those of their affiliated organizations, or those of the publisher, the editors and the reviewers. Any product that may be evaluated in this article, or claim that may be made by its manufacturer, is not guaranteed or endorsed by the publisher.

## References

[1] Din N Patel NN. Blepharitis—a review of diagnosis and management. Int J Ophthalmic Pract. (2012) 3:150–5. 10.12968/ijop.2012.3.4.150

[2] Bernardes TF Bonfioli AA. Blepharitis. Semin Ophthalmol. (2010) 25:79–83. 10.3109/08820538.2010.488562 20590417

[3] Peter V Kathryn C. Current evidence for topical azithromycin 1% ophthalmic solution in the treatment of blepharitis and blepharitis-associated ocular dryness. Int Ophthalmol Clin. (2011) 51:43–52. 10.1097/IIO.0b013e31822d6af1 21897139

[4] O'Brien TP. The role of bacteria in blepharitis. Ocul Surf. (2009) 7:S21–2. 10.1016/S1542-0124(12)70624-9 19445092

[5] Zhao YE Wu LP Hu L Xu JR. Association of blepharitis with demodex: a meta-analysis. Ophthalmic Epidemiol. (2012) 19:95–102. 10.3109/09286586.2011.642052 22364595

[6] Watters GA Turnbull PR Swift S Petty A Craig JP. Ocular surface microbiome in meibomian gland dysfunction. Clin Experiment Ophthalmol. (2017) 45:105–11. 10.1111/ceo.12810 27473509

[7] Dougherty JM McCulley JP. Comparative bacteriology of chronic blepharitis. Br J Ophthalmol. (1984) 68:524–8. 10.1136/bjo.68.8.524 6743618 PMC1040405

[8] Groden LR Murphy B Rodnite J Genvert GI. Lid flora in blepharitis. Cornea. (1991) 10:50–3. 10.1097/00003226-199110010-00010 2019106

[9] Bezza Benkaouha I Le Brun C Pisella PJ Chandenier J Lanotte P. La flore bactérienne dans les blépharites [Bacterial flora in blepharitis]. J Fr Ophtalmol. (2015) 38:723–8. 10.1016/j.jfo.2015.01.012 25982425

[10] Zhu M Cheng C Yi H Lin L Wu K. Quantitative analysis of the bacteria in blepharitis with demodex infestation. Front Microbiol. (2018) 9:1719. 10.3389/fmicb.2018.01719 30108572 PMC6079233

[11] Ozkan J Willcox MD. The ocular microbiome: molecular characterisation of a unique and low microbial environment. Curr Eye Res. (2019) 44:685–94. 10.1080/02713683.2019.1570526 30640553

[12] de Paula A Oliva G Barraquer RI de la Paz MF. Prevalence and antibiotic susceptibility of bacteria isolated in patients affected with blepharitis in a tertiary eye centre in Spain. Eur J Ophthalmol. (2020) 30:991–7. 10.1177/1120672119854985 31232091

[13] Dong Q Brulc JM Iovieno A Bates B Garoutte A Miller D. Diversity of bacteria at healthy human conjunctiva. Invest Ophthalmol Vis Sci. (2011) 52:5408–13. 10.1167/iovs.10-6939 21571682 PMC3176057

[14] Lee SH Oh DH Jung JY Kim JC Jeon CO. Comparative ocular microbial communities in humans with and without blepharitis. Invest Ophthalmol Vis Sci. (2012) 53:5585–93. 10.1167/iovs.12-9922 22836761

[15] Yan Y Yao Q Lu Y Shao C Sun H Li Y. Association between demodex infestation and ocular surface microbiota in patients with demodex blepharitis. Front Med. (2020) 7:592759. 10.3389/fmed.2020.592759 33251239 PMC7672197

[16] Dong X Wang Y Wang W Lin P Huang Y. Composition and diversity of bacterial community on the ocular surface of patients with meibomian gland dysfunction. Invest Ophthalmol Vis Sci. (2019) 60:4774–83. 10.1167/iovs.19-27719 31738825

[17] Amescua G Akpek EK Farid M Garcia-Ferrer FJ Lin A Rhee MK. Blepharitis preferred practice pattern®. Ophthalmology. (2019) 126:P56–93. 10.1016/j.ophtha.2018.10.019 30366800

[18] Lemp MA Nichols KK. Blepharitis in the United States 2009: a survey-based perspective on prevalence and treatment. Ocul Surf. (2009) 7:S1–14. 10.1016/S1542-0124(12)70620-1 19383269

[19] Jackson WB. Blepharitis: current strategies for diagnosis and management. Can J Ophthalmology. (2008) 43:170–9. 10.3129/i08-016 18347619

[20] Ozkan J Nielsen S Diez-Vives C Coroneo M Thomas T Willcox M. Temporal stability and composition of the ocular surface microbiome. Sci Rep. (2017) 7:9880. 10.1038/s41598-017-10494-9 28852195 PMC5575025

[21] Ozkan J Willcox M Wemheuer B Wilcsek G Coroneo M Thomas T. Biogeography of the human ocular microbiota. Ocul Surf. (2019) 17:111–8. 10.1016/j.jtos.2018.11.005 30445178

[22] Doan T Akileswaran L Andersen D Johnson B Ko N Shrestha A. Paucibacterial microbiome and resident DNA virome of the healthy conjunctiva. Invest Ophthalmol Vis Sci. (2016) 57:5116–26. 10.1167/iovs.16-19803 27699405 PMC5054734

[23] Huang Y Yang B Li W. Defining the normal core microbiome of conjunctival microbial communities. Clin Microbiol Infect. (2016) 22:643.e7–12. 10.1016/j.cmi.2016.04.008 27102141

[24] Suzuki T Sutani T Nakai H Shirahige K Kinoshita S. The microbiome of the meibum and ocular surface in healthy subjects. Invest Ophthalmol Vis Sci. (2020) 61:18. 10.1167/iovs.61.2.18 32053729 PMC7326502

[25] Kwon MS Lim SK Jang JY Lee J Park HK Kim N. Lactobacillus sakei WIKIM30 ameliorates atopic dermatitis-like skin lesions by inducing regulatory T cells and altering gut microbiota structure in mice. Front Immunol. (2018) 9:1905. 10.3389/fimmu.2018.01905 30154801 PMC6102352

[26] Chen J Yue Y Wang L Deng Z Yuan Y Zhao M. Altered gut microbiota correlated with systemic inflammation in children with Kawasaki disease. Sci Rep. (2020) 10:14525. 10.1038/s41598-020-71371-6 32884012 PMC7471315

[27] Lv Q Xu D Zhang X Yang X Zhao P Cui X. Association of hyperuricemia with immune disorders and intestinal barrier dysfunction. Front Physiol. (2020) 11:524236. 10.3389/fphys.2020.524236 33329010 PMC7729003

[28] Chen G Zhuang J Cui Q Jiang S Tao W Chen W. Two bariatric surgical procedures differentially alter the intestinal microbiota in obesity patients. Obes Surg. (2020) 30:2345–61. 10.1007/s11695-020-04494-4 32152837

[29] Markowiak P Slizewska K. Effects of probiotics, prebiotics, and synbiotics on human health. Nutrients. (2017) 9:1021. 10.3390/nu9091021 28914794 PMC5622781

[30] Guo X Zhang J Wu F Zhang M Yi M Peng Y. Different subtype strains of Akkermansia muciniphila abundantly colonize in southern China. J Appl Microbiol. (2016) 120:452–9. 10.1111/jam.13022 26666632 PMC4736461

[31] Belzer C de Vos WM. Microbes inside—from diversity to function: the case of Akkermansia. ISME J. (2012) 6:1449–58. 10.1038/ismej.2012.6 22437156 PMC3401025

[32] Johansson ME Hansson GC. Immunological aspects of intestinal mucus and mucins. Nat Rev Immunol. (2016) 16:639–49. 10.1038/nri.2016.88 27498766 PMC6435297

[33] Wang L Cui H Li Y Cao M Man S Guo L. Kang-Xian pills inhibit inflammatory response and decrease gut permeability to treat carbon tetrachloride-induced chronic hepatic injury through modulating gut microbiota. Evid Based Complementary Altern Med. (2020) 2020:8890182. 10.1155/2020/8890182 33144872 PMC7596455

[34] Sears CL. Enterotoxigenic Bacteroides fragilis: a rogue among symbiotes. Clin Microbiol Rev. (2009) 22:349–69. 10.1128/CMR.00053-08 19366918 PMC2668231

[35] Ryan MP Adley CC. Ralstonia spp.: emerging global opportunistic pathogens. Eur J Clin Microbiol Infect Dis. (2014) 33:291–304. 10.1007/s10096-013-1975-9 24057141

[36] Zhang-Sun W Tercé F Burcelin R Novikov A Serino M Caroff M. Structure function relationships in three lipids A from the Ralstonia genus rising in obese patients. Biochimie. (2019) 159:72–80. 10.1016/j.biochi.2019.01.015 30703476

[37] Zhang L Gowardman J Morrison M Krause L Playford EG Rickard CM. Molecular investigation of bacterial communities on intravascular catheters: no longer just Staphylococcus. Eur J Clin Microbiol Infect Dis. (2014) 33:1189–98. 10.1007/s10096-014-2058-2 24500600

[38] Wang Y Chen CH Hu D Ulmschneider MB Ulmschneider JP. Spontaneous formation of structurally diverse membrane channel architectures from a single antimicrobial peptide. Nat Commun. (2016) 7:13535. 10.1038/ncomms13535 27874004 PMC5121426

[39] Lobo SA Scott A Videira MA Winpenny D Gardner M Palmer MJ. Staphylococcus aureus haem biosynthesis: characterisation of the enzymes involved in final steps of the pathway. Mol Microbiol. (2015) 97:472–87. 10.1111/mmi.13041 25908396

[40] Zhao F Zhang D Ge C Zhang L Reinach PS Tian X. Metagenomic profiling of ocular surface microbiome changes in meibomian gland dysfunction. Invest Ophthalmol Vis Sci. (2020) 61:22. 10.1167/iovs.61.8.22 32673387 PMC7425691

[41] Deng Y Wen X Hu X Zou Y Zhao C Chen X. Geographic difference shaped human ocular surface metagenome of young han chinese from Beijing, Wenzhou, and Guangzhou cities. Invest Ophthalmol Vis Sci. (2020) 61:47. 10.1167/iovs.61.2.47 32106294 PMC7329964

[42] Skowron K Bauza-Kaszewska J Kraszewska Z Wiktorczyk-Kapischke N Grudlewska-Buda K Kwiecińska-Piróg J. Human skin microbiome: impact of intrinsic and extrinsic factors on skin microbiota. Microorganisms. (2021) 9:543. 10.3390/microorganisms9030543 33808031 PMC7998121

[43] Chen YE Tsao H. The skin microbiome: current perspectives and future challenges. J Am Acad Dermatol. (2013) 69:143–55. 10.1016/j.jaad.2013.01.016 23489584 PMC3686918

[44] Eom Y Na KS Hwang HS Cho KJ Chung TY Jun RM. Clinical efficacy of eyelid hygiene in blepharitis and meibomian gland dysfunction after cataract surgery: a randomized controlled pilot trial. Sci Rep. (2020) 1:11796. 10.1038/s41598-020-67888-5 32678131 PMC7366917

[45] Nowomiejska K Lukasik P Brzozowska A Toro MD Sedzikowska A Bartosik K. Prevalence of ocular demodicosis and ocular surface conditions in patients selected for cataract surgery. J Clin Med. (2020) 10:3069. 10.3390/jcm9103069 32977656 PMC7598293

[46] Miño de Kaspar H Shriver EM Nguyen EV Egbert PR Singh K Blumenkranz MS. Risk factors for antibiotic-resistant conjunctival bacterial flora in patients undergoing intraocular surgery. Graefe's Arch Clin Exp Ophthalmol. (2003)9:730–3. 10.1007/s00417-003-0742-5 12928904

[47] Miño De Kaspar H Ta CN Froehlich SJ Schaller UC Engelbert M Klauss V. Prospective study of risk factors for conjunctival bacterial contamination in patients undergoing intraocular surgery. Eur J Ophthalmol. (2009)5:717–22. 10.1177/112067210901900505 19787587

