# Supplementary material for: Composition and Diversity of the Ocular Surface Microbiota in Patients With Blepharitis in Northwestern China
Source: Front Med (Lausanne). 2021 Dec 7;8:768849. doi: 10.3389/fmed.2021.768849 (PMC8688757; doi:10.3389/fmed.2021.768849)
Supplement: Supplementary file 1 [file Data_Sheet_1.pdf]

Supplementary Material

DNA Extraction,

DNA was extracted from all samples using the MN NucleoSpin 96 Soi DNA Isolation Kit (MN-MACHEREY-NAGEL, Germany). The purity of bacterial genomic DNA was determined with a NanoDrop 2000 (Thermo Fisher Scientific, Waltham, USA) and the DNA integrity and quality were verified by 1.0% agarose gel electrophoresis. Then DNA was stored at -80°C until further processing.

PCR Amplification and 16S rDNA Amplicon Sequencing

The V3-V4 hypervariable region of the 16S rRNA gene was amplified with the common primer pair (Forward primer, 5’- ACTCCTACGGGAGGCAGCA-3’; Reverse primer, 5’-GGACTACHVGGGTWTCTAAT-3’) combined with adapter sequences and barcode sequences. PCR amplification was performed in a total volume of 10 μl, which contained 50 ng genome DNA, 0.3 μl Vn F (10 μM), 0.3 μl Vn R (10 μM), 5 μl KOD FX Neo Buffer, 2 μl dNTP (2 mM each), 0.2 μl KOD FX Neo, and the ddH2O increased to 10 μl. Thermal cycling conditions were as follows: an initial denaturation at 95 °C for 5 min, followed by 25 cycles at 95 °C for 30 s, 50 °C for 30 s and 72 °C for 40 s, with a final extension at 72 °C for 7 min. The PCR products from the first step PCR were purified through VAHTSTM DNA Clean Beads. A second round PCR was then performed in a 20μl reaction which contained 10 μl 2×Q5 HF MM, 2.5 μl MPPI-a (2 μM), 2.5 μl MPPI-b (2 μM) and 5μl PCR products from the first step. Thermal cycling conditions were as follows: an initial denaturation at 98 °C for 30s, followed by 10 cycles at 98 °C for 10s, 65 °C for 30s and 72 °C for 30s, with a final extension at 72 °C for 5 min. Finally, the PCR products were quantified by 1.8% agarose gel electrophoresis (ImageJ software), and the samples were mixed according to the mass ratio at 1:1. After mixing the samples, the samples were purified by OMEGADNA column and were recovered using a 1.8% agarose gel electrophoresis.

High-throughput sequencing analysis of bacterial rRNA genes was performed on the purified, pooled sample using the Illumina Hiseq 2500 platform (2×250 paired ends) at Biomarker Technologies Corporation, Beijing, China.

2.5 Bioinformatics and Statistical Analysis

Bioinformatics analysis was conducted on the BMK Cloud Platform (www.biocloud.net). The raw reads were filtered by Trimmomatic (version 0.33), and then, Cutadapt (version 1.9.1), FLASH (version 1.2.7) and UCHIME (version 4.2) were used to obtain the effective reads. The effective reads with more than 97% identity were clustered into operational taxonomic units (OTUs) by USEARCH (version 10.0), while the OTUs whose proportions were less than 0. 005% of the total OTUs were removed. Taxonomy was assigned using Silva (http://www.arb-silva.de) as the reference database, and the community composition of each sample was calculated at various levels (phylum, class, order, family, genus and species). The alpha- and beta- diversities of all groups were obtained using QIIME2 and QIIME software respectively. Linear discriminant analysis effect size (LEfSe) was used to identify bacterial biomarkers of each group. Student’s t-test was used to compare the differences in age and sex between the patients with blepharitis and the controls. The Mann-Whitney U-test was performed for analyses of the α-diversity indices and the relative abundances of dominant phyla and genera among different groups. For principle coordinate analysis (PCoA), the permutational multivariate analysis of variance (PERMANOVA) statistical method was used to

compare the differences. Statistical analyses were carried out with SPSS 26.0 (Chicago, IL, USA) software, and $p < 0.05$ was considered to be statistically significant.
